# Extraterrestrial artificial photosynthetic materials for *in-situ* resource utilization

**DOI:** 10.1093/nsr/nwab104

**Published:** 2021-06-12

**Authors:** Liuqing Yang, Ce Zhang, Xiwen Yu, Yingfang Yao, Zhaosheng Li, Congping Wu, Wei Yao, Zhigang Zou

**Affiliations:** Eco-Materials and Renewable Energy Research Center (ERERC), Jiangsu Key Laboratory for Nano Technology, National Laboratory of Solid State Microstructures, School of Physics, Nanjing University, Nanjing 210093, China; Qian Xuesen Laboratory of Space Technology, China Academy of Space Technology, Beijing 100094, China; Eco-Materials and Renewable Energy Research Center (ERERC), Jiangsu Key Laboratory for Nano Technology, National Laboratory of Solid State Microstructures, School of Physics, Nanjing University, Nanjing 210093, China; Collaborative Innovation Center of Advanced Microstructures, College of Engineering and Applied Sciences, Nanjing University, Nanjing 210093, China; Eco-Materials and Renewable Energy Research Center (ERERC), Jiangsu Key Laboratory for Nano Technology, National Laboratory of Solid State Microstructures, School of Physics, Nanjing University, Nanjing 210093, China; Collaborative Innovation Center of Advanced Microstructures, College of Engineering and Applied Sciences, Nanjing University, Nanjing 210093, China; KunshanInnovation Institute of Nanjing University, Suzhou 215347, China; School of Science and Engineering, The Chinese University of Hong Kong, Shenzhen 518172, China; Wuhan National Laboratory for Optoelectronics, Huazhong University of Science and Technology, Wuhan 430074, China; Eco-Materials and Renewable Energy Research Center (ERERC), Jiangsu Key Laboratory for Nano Technology, National Laboratory of Solid State Microstructures, School of Physics, Nanjing University, Nanjing 210093, China; Collaborative Innovation Center of Advanced Microstructures, College of Engineering and Applied Sciences, Nanjing University, Nanjing 210093, China; Eco-Materials and Renewable Energy Research Center (ERERC), Jiangsu Key Laboratory for Nano Technology, National Laboratory of Solid State Microstructures, School of Physics, Nanjing University, Nanjing 210093, China; KunshanInnovation Institute of Nanjing University, Suzhou 215347, China; Qian Xuesen Laboratory of Space Technology, China Academy of Space Technology, Beijing 100094, China; Eco-Materials and Renewable Energy Research Center (ERERC), Jiangsu Key Laboratory for Nano Technology, National Laboratory of Solid State Microstructures, School of Physics, Nanjing University, Nanjing 210093, China; Qian Xuesen Laboratory of Space Technology, China Academy of Space Technology, Beijing 100094, China; Collaborative Innovation Center of Advanced Microstructures, College of Engineering and Applied Sciences, Nanjing University, Nanjing 210093, China; School of Science and Engineering, The Chinese University of Hong Kong, Shenzhen 518172, China; Macau Institute of Systems Engineering, Macau University of Science and Technology, Macau 999078, China

**Keywords:** solar energy, extraterrestrial survival, artificial photosynthesis, CO_2_ reduction, oxygen evolution

## Abstract

Aerospace milestones in human history, including returning to the moon and manned Martian missions, have been implemented in recent years. Space exploration has become one of the global common goals, and to ensure the survival and development of human beings in the extraterrestrial extreme environment has been becoming the basic ability and technology of manned space exploration. For the purpose of fulfilling the goal of extraterrestrial survival, researchers in Nanjing University and the China Academy of Space Technology proposed extraterrestrial artificial photosynthesis (EAP) technology. By simulating the natural photosynthesis of green plants on the Earth, EAP converts CO_2_/H_2_O into fuel and O_2_ in an *in-situ*, accelerated and controllable manner by using waste CO_2_ in the confined space of spacecraft, or abundant CO_2_ resources in extraterrestrial celestial environments, e.g. Mars. Thus, the material loading of manned spacecraft can be greatly reduced to support affordable and sustainable deep space exploration. In this paper, EAP technology is compared with existing methods of converting CO_2_/H_2_O into fuel and O_2_ in the aerospace field, especially the Sabatier method and Bosch reduction method. The research progress of possible EAP materials for *in-situ* utilization of extraterrestrial resources are also discussed in depth. Finally, this review lists the challenges that the EAP process may encounter, which need to be focused on for future implementation and application. We expect to deepen the understanding of artificial photosynthetic materials and technologies, and aim to strongly support the development of manned spaceflight.

## Teaser

The extraterrestrial artificial photosynthesis *in situ* converts CO_2_ in spacecraft's confined space and in Martian atmosphere into O_2_ and fuels, thus supporting affordable and sustainable deep-space exploration and migration plans.

## INTRODUCTION

Extraterrestrial survival is a prerequisite for humankind to achieve long-term space flight, extraterrestrial residence and immigration, and creates one of the greatest scientific and technological challenges of human deep space exploration. During human extraterrestrial exploration activities, a sustained supply of O_2_ and fuel is one of the essential abilities. Extraterrestrial artificial photosynthesis (EAP), i.e. CO_2_/H_2_O conversion into fuel and O_2_ from human respiration, combustion emissions and *in-situ* resources on outer-Earth planets, can greatly reduce the supply load of spacecraft and space stations, thus promoting affordable and sustainable human deep space exploration.

**Figure 1.**
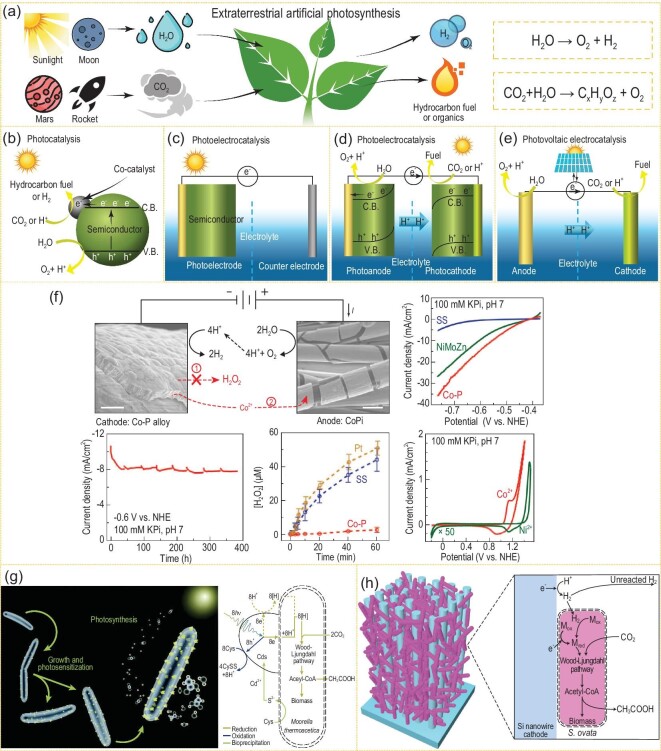
Mechanism schemes of (a) EAP, (b) photocatalysis, (c and d) photoelectrocatalysis and (e) photovoltaic electrocatalysis. (f) A biocompatible inorganic water-splitting catalyst system (adapted from ref. [16] with permission from American Association for the Advancement of Science (AAAS)). (g) Scheme of CO_2_ reduction of the bacteria/semiconductor hybrid artificial photosynthetic system (adapted from ref. [17] with permission from AAAS). (h) Illustrations of a nanowire-bacteria hybrid system and the reaction mechanism (adapted from ref. [18] with permission from Elsevier).

EAP (Fig. 1a) is a simulation of plant photosynthesis on Earth. Through photocatalysis [1,2] (Fig. 1b), photoelectrocatalysis [3,4] (Fig. 1c and d) or photovoltaic electrocatalysis [5] (Fig. 1e), EAP uses a controllable and accelerated chemical process to *in-situ* convert CO_2_/H_2_O into carbon-containing fuel and O_2_ by harnessing solar radiation. Compared with traditional CO_2_/H_2_O conversion techniques, such as the thermochemical or electrochemical method, EAP technology, which only uses solar energy and semiconductor materials, is usually carried out without consuming auxiliary energy inputs. EAP can be applied to *in-situ* convert CO_2_ waste in a confined space, which effectively reduces the supply demand of human space stations and deep space spacecraft, etc. Furthermore, it also makes use of abundant *in-situ* resources such as CO_2_ and H_2_O in the extraterrestrial atmospheric environments of the moon or Mars to meet the material demand. Through EAP, human beings can survive in extraterrestrial environments in the long term.

In recent decades, for solving the key problems of supply demand for human space stations and deep space exploration, the USA, Japan and other countries have continuously carried out research on CO_2_ conversion technology as one of the crucial parts of *in-situ* resource utilization, developed a series of CO_2_ conversion systems based on the Sabatier method [6] and Bosch reduction method [7], and carried out experimental verification of CO_2_ reduction and O_2_ production on the International Space Station (ISS) [8,9]. In 2005, the National Aeronautics and Space Administration (NASA) in the USA proposed the Resource Prospector mission, planning to carry out *in-situ* utilization experiments for producing O_2_ from water in lunar surface soil in 2022. Russia raised a series of plans for lunar exploration and will launch a lander named Moon 27 in 2025 for *in-situ* lunar resource utilization. However, existing CO_2_ conversion devices adopting high-temperature and high-pressure reaction conditions always have high energy consumption. On the other hand, the extraterrestrial microgravity environment obviously promotes the formation of supersaturated layers of dissolved gas molecules near the electrode surface. These layers accelerate the formation and evolution of bubbles at the interface between electrode and electrolyte, and hinder the material transport rate at the microscopic scale [10–12]. The electrode reaction kinetics are thus significantly reduced, resulting in less than one-third of the working efficiency on the Earth. Furthermore, due to the lack of experimental data and related theoretical research, key scientific and technical challenges, such as *in-situ* preparation of photoelectrocatalytic materials, the heterogeneous catalytic process and the working parameters of materials and systems, have become major problems in CO_2_ conversion.

The ability to harvest light energy through artificial photosynthesis may create an essential foundation for technologies used in many areas, such as global carbon neutralization [13]. Thus, it is reasonable to expect the further development of EAP for human extraterrestrial survival. With regard to this, one example is demonstrated by an EAP device, developed by Qian Xuesen Laboratory of Space Technology, which reduces CO_2_ with water into a carbonaceous compound and produces O_2_. The feasibility of the reactor in CO_2_/H_2_O photo-conversion into carbonaceous compound and O_2_ was verified by ground experiment, which may provide a theoretical and practical foundation for subsequent device optimization, carbon dioxide conversion into variable hydrocarbon products with high selectivity, and in-orbit testing of artificial photosynthesis devices [14].

The research into artificial photosynthesis began in 1972 when Honda and Fujishima reported that H_2_ was produced by photolysis of water over titanium dioxide electrodes under ultraviolet light [4]. Then during the following few decades, numerous scientists carried out a series of research works on this specific area. In 2001, Zhigang Zou proposed a new theory and method to regulate the band structure of photocatalytic materials, and broadened the response range of photocatalytic materials. He realized visible-light-induced complete water decomposition [2] and CO_2_ reduction [15] under visible light, thus developing a new-generation visible-light-responsive photocatalytic material. In 2015, NASA started to focus on artificial photosynthesis and proposed the concept of microbial-assisted artificial photosynthesis. In 2016, Daniel G. Nocera devised a biocompatible-inorganic catalyst system to decompose water to get H_2_ and O_2_ at low voltages [16] (Fig. 1f). He utilized low concentrations of CO_2_ in the presence of O_2_ and H_2_ to generate biomass, fuel or chemical products. A 10% energy efficiency of CO_2_ reduction could be obtained when coupling this device with a photovoltaic system. Peidong Yang combined light-absorbing semiconductor nanomaterials with bacteria to produce biological-inorganic hybrid systems for CO_2_ fixation [17,18] (Fig. 1g and h). In 2017, Nanjing University and Qian Xuesen Laboratory at the China Academy of Space Technology carried out research into EAP materials and systems for the first time, aiming to resolve the requirements with regard to basic materials and energy during human extraterrestrial survival, and they have preliminarily completed the verification of materials and systems. This research progress provides the technical foundation for human survival during spaceflight missions and deep space exploration.

## EXTRATERRESTRIAL ENVIRONMENTAL IMPACT

Space exploration activities are faced with various special environments, such as microgravity, strong radiation, extreme temperature and high vacuum, which bring about a series of challenges for realizing CO_2_/H_2_O conversion in outer space. Only through long-term and effective experiments, combined with ground simulation and in-orbit verification, can we investigate the mechanistic and process influences of outer space on EAP in the process of space exploration. However, it can be speculated that the external factors that mainly affect CO_2_ and H_2_O conversion may be, and are not limited to, the following aspects:

- Microgravity. Under microgravity, the key problems of bubble formation, evolution and detachment at the reaction interface need to be solved urgently.
- Cosmic radiation. Materials, especially semiconductors, on the lunar surface or in Earth orbit would be strongly affected by the impact of rays or particles such as electrons, protons, heavy ions and plasma. Material properties will be changed by electromagnetic radiation and charged particles, including X-rays, electrons, protons and heavy ions, mainly due to the internal interactions in materials during the bombardment caused by cosmic rays or high energy particles. These internal interactions are divided into coulomb interactions and electromagnetic effects.
  Coulomb interactions include three cases: Coulomb scattering, bremsstrahlung and inelastic collision between particles and electrons. Coulomb scattering refers to the elastic collision process in which charged particles are incident to matter, then deflected and dispersed by the Coulomb electric field force of the atomic nucleus. When materials are incident by high-energy electrons, the high-speed electron suddenly slows down and produces the bremsstrahlung, which is an important process for charged particles to act on materials in space. When particles or electrons inelastically collide with material, the electron in the material is ionized or excited from the outer layer to the inner layer, causing primary ionization to produce a large number of secondary electrons. The total kinetic energy before and after collision is not equal.
  Electromagnetic interaction mainly refers to the interaction between high-energy rays and materials. The whole, or part, of the initial energy of the high-energy ray can be transferred to electrons in the materials, and the incident particles disappear or scatter. Electromagnetic effects include the photoelectric effect, electron pair effect and Compton effect. The dominant effect of electromagnetic action is related to photon energy and atomic coefficient of absorbing material.
  After the incident particles enter the material, the energy of the incident particles decreases and the velocity slows down. Finally, the incident particles are blocked or scattered. The charged particles enter the material and lose energy through two ways: the displacement effect and ionization effect. The displacement effect is when charged particles collide with the nucleus, making atoms leave their original positions; or the incident particles fill in the lattice gaps to form vacancies and interstitial atoms, leading to the corresponding changes in material structure and properties. Collision between incident high-energy particles and material atoms is the main source of energy loss. Further, a large number of recoil atoms are produced after collision, and the secondary reactions of recoil atoms with the surrounding atoms form a large number of Frenkel defects. Most of these defects are semi-permanent, leading to great damage to semiconductor materials and devices. The ionization effect is when the radiation of charged particles with a certain energy excites electrons outside the nucleus of materials to form free electrons. Material atoms thus become positive ions, forming electron-hole pairs. When electrons transition from valence band to conduction band, the electrical, chemical, physical and mechanical properties of materials can be affected. Small-dose and long-term steady-state radiation in space often leads to a cumulative ionization damage effect.
- Extreme temperature. Extraterrestrial space, including the lunar surface, produces a huge temperature difference between day and night. During daytime, when the sun shines vertically, the temperature rises as high as 127ºC; at night, the temperature can be as low as −183ºC. As water evaporates or freezes easily, this poses a great challenge to CO_2_ reduction in aqueous systems. Furthermore, the thermal expansion and contraction caused by temperature switching generally accelerates the fatigue and aging of materials, which brings a series of system durability and reliability problems.

In addition, there are other problems caused by the atmospheric pressure and special atmospheric environments of extraterrestrial planets, although some of them, e.g. extreme temperatures and ultra-vacuums, can be resolved by aerospace engineering methods. For example, the Environmental Control and Life Support System (ECLSS) used on the ISS by NASA can maintain the space capsule pressure, temperature and humidity. Extreme conditions have brought great challenges for researchers in selecting and designing materials, and it will also become difficulties in our research of extraterrestrial artificial photosynthsis.

## RECENT PROGRESS ON EXTRATERRESTRIAL CO_2_ AND H_2_O CONVERSION FOR SPACECRAFT

**Figure 2.**
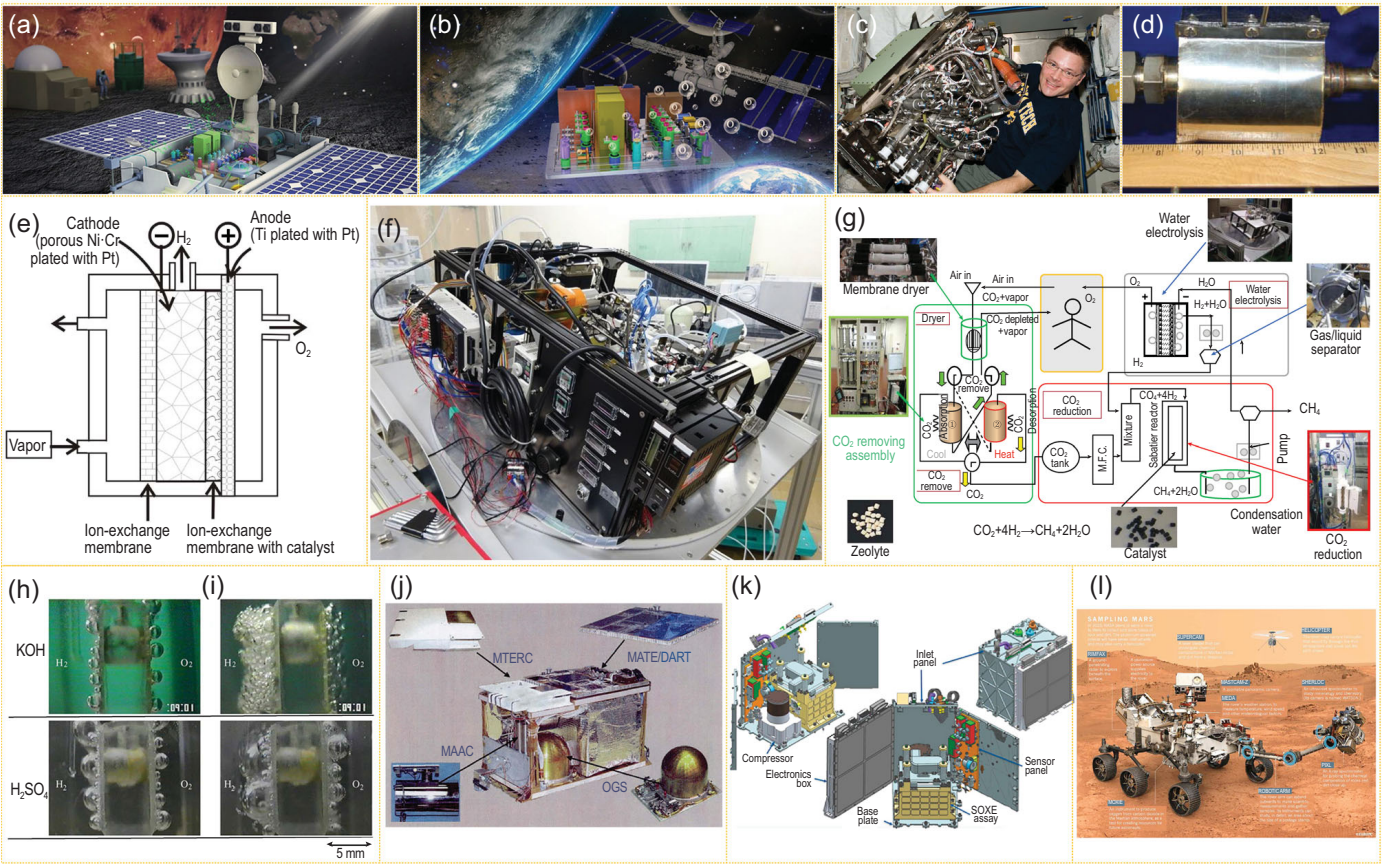
(a) Space exploration experiment device using *in-situ* resources to produce O_2_. (b) Partial enlargement of (a). (c) Sabatier reaction system loaded into the ISS (adapted from ref. [6] with permission from American Institute of Aeronautics and Astronautics (AAIA)). (d) The core unit of the reactor (adapted from ref. [20] with permission from AIAA). (e) The principle of the reactor (adapted from ref. [21] with permission from AIAA) and (f) exterior feature of the JAXA water electrolyzer (adapted from ref. [9] with permission from AIAA). (g) New conceptual ECLSS in space station (adapted from ref. [9] with permission from AIAA). (h) Comparison of electrode in the environments of normal gravity on Earth (adapted from ref. [10] with permission from Elsevier). (i) Microgravity in space during the water electrolyzing process (adapted from ref. [11] with permission from Elsevier). (j) MIP CO_2_ converting system (adapted from ref. [22] with permission from AIAA). (k) MOXIE CO_2_ converting system (adapted from ref. [24] with permission from Elsevier). (l) Set-up diagram of ‘Mars 2020’ (adapted from ref. [24] with permission from Elsevier).

Since the 1960s, the Sabatier method [6] and Bosch reduction method [7] have been the main approaches in CO_2_ reduction technology. H_2_O electrolysis for H_2_ and O_2_ has also been widely anticipated [19]. Recently, the EAP technology proposed by Nanjing University and Qian Xuesen Laboratory realized CO_2_/H_2_O photo-conversion under mild conditions with low energy consumption (Fig. 2a and b).

For solving the key problems of manned space stations and deep space exploration, the USA and other countries carried out research on CO_2_ and H_2_O conversion based on traditional ground technology, e.g. by using H_2_O electrolysis to supply O_2_ for astronauts in the ISS. To realize the recycling of CO_2_ released by astronauts, NASA and Japan Aerospace Exploration Agency (JAXA) have developed a set of CO_2_ reduction and O_2_ evolution devices, in which CO_2_ reduction is obtained by converting CO_2_ and H_2_ into methane with H_2_ obtained by H_2_O electrolysis. The Sabatier reactor contains a gas-solid two-phase process with a core unit temperature of 250–450ºC and a minimum gas pressure of 55 kPa. The mass of the ground experimental unit is around 41 kg and the total power is >100 W. The in-orbit test was completed for this system in October 2010 (Fig. 2c and d) [6,20]. The water electrolyzer developed by JAXA has also been tested in orbit [8,9]. This device was obtained by modifying the more technically mature proton exchange membrane electrolytic cell, which consists of an electrolytic unit and a gas-liquid separation unit [9,21] (Fig. 2e and f). In the ISS, a combination of the above two devices was tested to support the ECLSS to convert CO_2_ into O_2_ and methane by in-orbit reaction (Fig. 2g) [9]. JAXA researchers used parabolic flight and drop tower tests to make a series of research and improvement works on the water electrolysis device, including tests on the working temperature of the electrolytic cells, the pressure of the gas-liquid separation membranes, the electrolyte component, and the working voltage and current. However, even after various optimizations, the water electrolytic device’s efficiency under microgravity was less than a third of that under usual gravity environments. The supersaturated layers of dissolved gas molecules formed by the aggregation of over-dissolved gas on the electrode highly hindered the transport rate and reaction efficiency of electrolyte [10] (Fig. 2h and i). Matsushima *et al*. found that the interaction between electrodes and electrolytes in the microgravity environment has a significant impact on the formation and evolution of bubbles, and the electrolytic performance [12]. In order to improve the material transport rate and reaction efficiency under microgravity, Nanjing University and Qian Xuesen Laboratory used liquid shearing force to compel the generated gas from the electrode surface, to prevent bubble gathering near the electrode surface under microgravity conditions [14].

For the more challenging manned deep space exploration missions, the USA first proposed the scheme of producing O_2_ and fuel by using *in-situ* resources such as water and carbon dioxide on the moon or Mars. NASA proposed a Mars *in-situ*-propellant-production precursor (MIP) plan in 2001 to deoxidize carbon dioxide into O_2_ using high-temperature electrolysis (Fig. 2j) [22]. In 2013, NASA also proposed a Mars *in-situ* resource utilization landing mission, MARCO POLO [23], which would utilize Mars’s atmospheric and soil resources to produce H_2_, O_2_ and CH_4_ by the Sabatier method and water electrolytic technology. They further proposed the Mars Oxygen ISRU Experiment (MOXIE) load in 2014 to deoxidize carbon dioxide in the Martian atmosphere to generate O_2_ with a solid oxide electrolytic cell at 800ºC to achieve 10 g h^–1^ O_2_ production (Fig. 2k) [24]. The load project was launched in 2020, with about 2 h of experiments on Mars. If this *in-situ* resource utilization technology is validated, NASA will plan to follow up with a 100-fold magnificated scale device to support the 2033 manned Mars mission. In 2018, NASA supported a plan named the CO_2_ Conversion Challenge to develop novel synthesis technologies that use carbon dioxide to generate molecules that can be used to manufacture a variety of products. However, the selected projects have remained at the laboratory stage and do not show feasibility for application to spacecraft. The USA is extracting O_2_ from the Martian atmosphere as part of the ‘Mars 2020’ rover project (Fig. 2l) [24]. Generally, the American space mission in CO_2_ utilization and transformation mainly uses the relatively mature thermal or electrical chemical conversion technology found in industry. Although the technical route has high maturity and stability, it needs to be carried out under extremely high temperature conditions (900–1600ºC), with harsh operating conditions and large energy consumption, which is not conducive to manned deep space exploration. In 2020, Qian Xuesen Laboratory developed a demo of an EAP device for reducing carbon dioxide with water into carbonaceous compound and producing O_2_ [14]. The feasibility of the reactor in reducing carbon dioxide to O_2_ and carbonaceous compound was verified by ground experiment, which may provide a theoretical and practical foundation for subsequent device optimization, carbon dioxide conversion to variable hydrocarbon products with high selectivity, and on-orbit testing of artificial photosynthesis devices.

## MATERIALS FOR EAP

Even on the moon or Mars, the energy provided by the sun is considerable. The light intensity on the moon approximates 1.4 times that of the Earth. Recent research shows that the permanent light area on the moon is adjacent to the ice area, which may be an ideal place for human beings to set up bases on the moon [25]. Solar power on Mars’s surface is nearly 40% of that found on Earth. Because there is no atmospheric absorption, the solar spectrum on the surface of the moon is similar to that in Earth orbit, which is about equal to AM0 (1366.1 W m^–2^). The transmitted sunlight wavelength on the surface of Mars increases with the increase of solar zenith angle and optical depth, and the energy at shorter wavelengths is more easily exhausted, because the cross section of dust particles suspended in the atmosphere at shorter wavelengths is larger. The 60-degree spectrum of sunlight on the surface of Mars is reduced to one-sixth of AM0, and further reduced in the blue part of the spectrum [26]. The moon has no atmosphere, thus there is no CO_2_ on the moon. It is conservatively estimated that 0.3% to 1% of the water on the moon is buried in the form of ice under 40 cm of dry regolith [27]. On Mars, 95% of the atmosphere (the average pressure is <1% of one atmosphere) is CO_2_. It has been estimated that Martian meteorites contain carbonates in low abundances (<1 vol.%) [28], and calcium carbonate has been identified in the soils at the Mars Phoenix landing site [29]. Thus, it is important to evaluate the feasibility of utilizing such mineralized carbon in

future research. At this stage this review mainly focuses on the EAP process of CO_2_/H_2_O. Moreover, ground ice and hydration water exist at the near-surface subsurface of Mars [27]. The above preconditions are propitious to EAP in extraterrestrial environments such as the moon and Mars.

Most of the practical applications of EAP are based on photocatalytic materials. Photocatalysts are an important component to support artificial photosynthesis. Catalysts suitable for EAP are herein classified as photocatalysts, photoanodes, photocathodes and photovoltaic-electrochemical catalysts. The evaluation parameters for the performance assessment of the above catalysts mainly contain product rate, catalytic current density (for photoelectrocatalysts), turnover number and turnover frequency (evaluating the activity of catalytic active sites), quantum yield (assessing the performance of photocatalysts) and faradaic efficiency (assessing the selectivity for photoelectrocatalysts). In the following text, we will mainly analyze photocatalytic/photoelectrocatalytic materials for EAP.

EAP processes mainly involve the conversion of CO_2_ and H_2_O into hydrocarbon fuel and O_2_, and water splitting to produce H_2_ and O_2_. CO_2_/H_2_O photo-conversion involves two parts of the half reaction, including CO_2_ reduction by photo-generated electrons, and H_2_O oxidation by photo-generated holes, respectively. The standard chemical potentials required in thermodynamics for these processes are shown in the following reactions (1–8), respectively:

(1) \begin{equation*} 2{{\rm{H}}_2}{\rm{O}} + 2{{\rm{e}}^-} \to {{\rm{H}}_2}\ \quad \left( {{\rm{E}} = 0\ {\rm{V}}\,\,{\rm{vs}}. {\rm{\,SHE}}} \right), \end{equation*}

(2) \begin{eqnarray*} 2{{\rm{H}}_2}{\rm{O}} + 4{{\rm{h}}^ + } \to 4{{\rm{H}}^ + } + {{\rm{O}}_2} \nonumber\\ &&\quad \left( {{\rm{E}} = + 1.23\ {\rm{V}}\,\,{\rm{vs}}. {\rm{\,SHE}}} \right),\end{eqnarray*}

(3) \begin{eqnarray*} {\rm{C}}{{\rm{O}}_2} + {{\rm{e}}^-} \to {\rm{C}}{{\rm{O}}_2}{\cdot^-}\nonumber\\ &&\qquad \left( {{\rm{E}} = - 1.90\ {\rm{V}}\,{\rm{vs}}.\,{\rm{SHE}}} \right),\end{eqnarray*}

(4) \begin{eqnarray*} {\rm{C}}{{\rm{O}}_2} + 2{{\rm{H}}^ + } + 2{{\rm{e}}^-} \to {\rm{CO}} + {{\rm{H}}_2}{\rm{O}}\nonumber\\ &&\qquad \left( {{\rm{E}} = - 0.53\ {\rm{V}}\,\,{\rm{vs}}.\,\,{\rm{SHE}}} \right),\end{eqnarray*}

(5) \begin{eqnarray*} {\rm{C}}{{\rm{O}}_2} + 2{{\rm{H}}^ + } + 2{{\rm{e}}^-} \to {\rm{HCOOH}}\nonumber\\ &&\qquad \left( {{\rm{E}}\,\, = \,\, - 0.61\ {\rm{V}}\,\,{\rm{vs}}.\,\,{\rm{SHE}}} \right),\end{eqnarray*}

(6) \begin{eqnarray*} {\rm{C}}{{\rm{O}}_2} + 4{{\rm{H}}^ + } + 4{{\rm{e}}^-} \to {\rm{HCHO}} + {{\rm{H}}_2}{\rm{O}}\nonumber\\ &&\qquad \left( {{\rm{E}}\,\, = \,\, - 0.48\ {\rm{V}}\,\,{\rm{vs}}.\,\,{\rm{SHE}}} \right),\end{eqnarray*}

(7) \begin{eqnarray*} {\rm{C}}{{\rm{O}}_2} + 6{{\rm{H}}^ + } + 6{{\rm{e}}^-} \to {\rm{C}}{{\rm{H}}_3}{\rm{OH}} + {{\rm{H}}_2}{\rm{O}}\nonumber\\ &&\qquad \left( {{\rm{E}} = - 0.38\ {\rm{V}}\,\,{\rm{vs}}.\,\,{\rm{SHE}}} \right),\end{eqnarray*}

(8) \begin{eqnarray*} {\rm{C}}{{\rm{O}}_2} + 8{{\rm{H}}^ + } + 8{{\rm{e}}^-} \to {\rm{C}}{{\rm{H}}_4} + 2{{\rm{H}}_2}{\rm{O}}\nonumber\\ &&\qquad \left( {{\rm{E}} = - 0.24\ {\rm{V}}\,\,{\rm{vs}}.\,\,{\rm{SHE}}} \right).\end{eqnarray*}

In consideration of the extreme environmental conditions that EAP materials would be applied in, EAP materials should meet the following requirements: (i) an appropriate band structure, which is conducive for extraterrestrial sunlight absorption with different spectra and higher intensity, and reaches the chemical potentials needed to achieve the corresponding photo-redox reaction; (ii) adequate surface catalytic active sites to support effective photo-redox reaction; (iii) fast carrier transport and separation at the interface; (iv) stable activity under microgravity; (v) excellent survivability under intense cosmic radiations; (vi) great capability of resisting impact during take-off and landing; (vii) low cost. Additionally, CO_2_/H_2_O photocatalysts require excellence in reactant adsorption, product desorption and product selectivity. During CO_2_/H_2_O photo-conversion, H_2_ production is the major competitive side reaction. Therefore, avoiding H_2_ production is also one of the key points in improving CO_2_/H_2_O photo-conversion. A major way to hinder the H_2_ production is to adjust the surface adsorption behavior of protons during the EAP process. Typical methods [30] include adjusting adsorption properties of the catalyst surface on carbon intermediate species, realizing proper surface modification and hydrating or alloying the surface with cocatalysts.

### EAP materials for overall photo-conversion

Most of the reported photocatalytic materials are thermodynamically unfavorable to complete both the reductive half reaction (CO_2_ reduction) and the oxidative half reaction (O_2_ production), since the overall CO_2_ conversion reaction generally needs a band gap of over 3.1 eV when taking account of the CO_2_ adsorption on the electrode surface (reaction (3)). Catalysts with suitable band structure supporting overall CO_2_/H_2_O conversion are mainly TiO_2_, ZnO, CdS, ZnS, ZnIn_2_S_4_, Ta_3_N_5_, TaON, graphitic carbon nitride (*g*-C_3_N_4_), SrTiO_3_ [31–35] (in which TiO_2_, ZnO and ZnS can only use ultraviolet light). SrTiO_3_ and CdS can theoretically carry out full conversion under visible light, however, their intrinsic properties are restricted by low carrier separation rate. Thus, modification by cocatalysts or construction of composite materials is needed for the practical application of overall CO_2_/H_2_O conversion. Most of the cocatalysts in photocatalysis are derived from electrocatalysts. The role of cocatalysts is to reduce the activation energy required for surface reactions. Cocatalysts for oxygen evolution reaction (OER) mainly include cobalt phosphate (CoPi) [36], iron hydroxide oxide (FeOOH) [37], nickel oxide (NiO_x_) [38], ceria (CeO_x_) [39], RuO_2_ [40], cobalt oxide (CoO_x_) [41] and manganese oxide (MnO_x_) [42]. Highly active cocatalysts for CO_2_ reduction reaction (CO_2_RR) primarily contain Au [43], Ag [44], Cu [45], Mn [46], MoS_2_ [47] and CdS [48]. Moreover, adjusting the composition and ratio of metals, metallic alloy or other composite cocatalysts can be helpful in improving the selectivity of CO_2_ reduction products [49–51].

At present, EAP materials are restricted by the low efficiency of photo-generated charge separation, the slow surface reaction rate and the serious inverse reaction of the products. A variety of methods have been developed to regulate the charge separation and surface reaction performance, as well as restrain the inverse reaction of the artificial photosynthetic catalysts, including crystal engineering, built-in electric field, polarization effect, effective mass reduction of photo-generated carriers, a single crystal with low structural defects, molecular composites, the Z-scheme strategy and surface modification with cocatalysts. Conformation of solid solution is one of the effective means of regulating the electronic structure of catalysts and promoting photosynthetic performance. Zhigang Zou’s group showed in their theoretical calculation results that Zn_2_GeO_4_ phase transformation from pseudo-cubic phase into cubic phase can effectively narrow the band gap. This is because the introduction of s and p orbitals of Ge enhances the repulsion of p-d (O 2p-Zn 3d) and raises the valence band position, while the s orbitals of Ge with low energy effectively lower the conduction band position. The solid solution photocatalysts composed of cubic ZnGa_2_O_4_ and pseudo-cubic Zn_2_GeO_4_ have light hole effective mass, and higher hole mobility. A narrow band gap is beneficial for absorbing sunlight with a wider wavelength, and high hole mobility is beneficial for improving the reaction rate of water oxidation. Therefore, the solid solution photocatalytic materials show higher photocatalytic performance with regard to reducing CO_2_ to hydrocarbon fuel [52]. Based on a similar mechanism, Kazunari Domen’s group also reported that (Ga_1−x_Zn_x_N_1−x_O_x_) has excellent performance for visible-light-driven complete water splitting [53].

**Figure 3.**
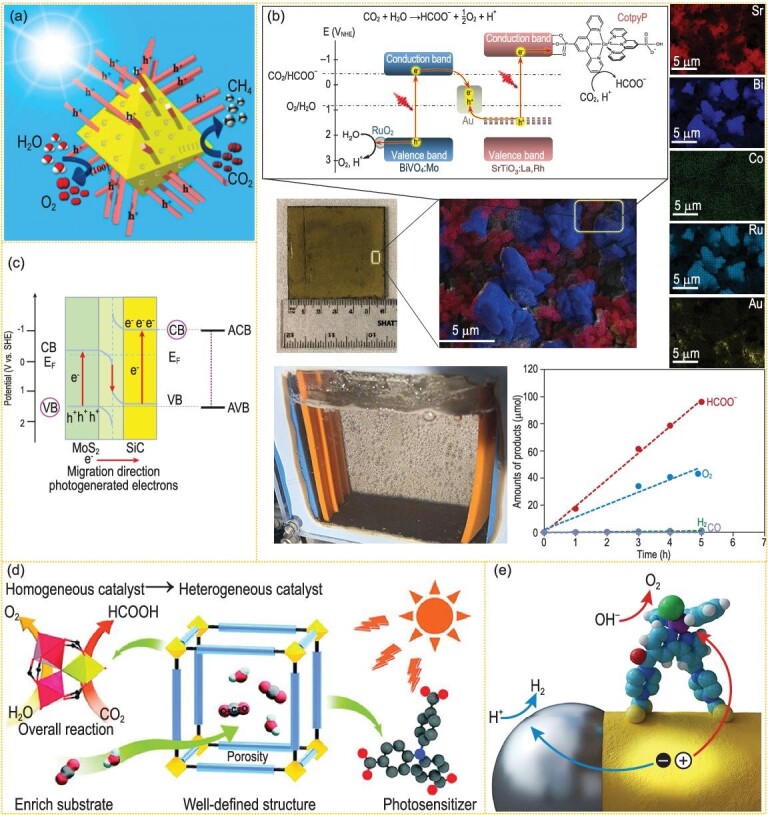
(a) Schematic illustration of CO_2_ photoreduction into CH_4_ over hexahedron prism anchored octahedronal CeO_2_ (adapted from ref. [54] with permission from ACS Publications). (b) An overall CO_2_/H_2_O photo-conversion system with the CotpyP-loaded SrTiO_3_:La,Rh|Au|RuO_2_-BiVO_4_:Mo photocatalysts (adapted from ref. [58] with permission from Springer Nature). (c) Sketch map of Z-scheme model and light-induced charge transfer path over SiC@MoS_2_ photocatalyst (adapted from ref. [59] with permission from ACS Publications). (d) CO_2_/H_2_O photo-conversion reaction route of the heterometallic cluster-based organic frame photocatalyst (adapted from ref. [60] with permission from Wiley-VCH). (e) Diagrammatic sketch of water splitting on Pt nanoparticle (gray) decorated CdS (yellow) (adapted from ref. [31] with permission from Springer Nature).

It is an effective method to separate photo-generated charges by forming a built-in electric field on the interface of materials. Commonly, the built-in electric field exists between two different semiconductor materials or between different phase structures of the same semiconductor. The electrons and holes in this type of built-in electric field separate to opposite directions under the electric field, thus improving the separation efficiency. Zhigang Zou *et al*. synthesized a CeO_2_ octahedral structure with vertical growth of hexahedron prism [54] (Fig. 3a). By adjusting the length and number of CeO_2_ prisms with exposed surface of {1 0 0} facets on octahedron with surface of {1 1 1} facets, the separation and transmission efficiency of photo-generated carriers were improved, leading to the highly improved efficiency of photocatalytic CO_2_ reduction to produce CH_4_. Theoretical calculation showed that for CeO_2_, the effective mass of electrons on {1 0 0} facet is much larger than that on {1 1 1} facet, while the effective mass of holes on {1 1 1} facet is larger than that on {1 0 0} facet. Therefore, photo-generated electrons easily migrate to the {1 1 1} facets, while photo-generated holes easily migrate to the {1 0 0} facets, which is conducive to the separation of photo-generated e^–^ and h^+^. Thus, the CeO_2_ homojunction materials achieved the overall CO_2_/H_2_O photo-conversion efficiency with 0.86 μmol h^–1^ g^–1^ CH_4_ yield, and 0.2% quantum yield at 380 nm. The O_2_ yield was also detected in accord with ∼2 : 1 molar ratio to CH_4_.

Polar semiconductors with asymmetric positive and negative electron centers can induce the polarization effect for effective separation of photo-generated charges. Zhigang Zou *et al*. proposed that single crystals growing along the polarization axis of a polar semiconductor could maximize the polarization field effect of the polar semiconductor, and efficiently separate photo-generated charges [55]. Because of the periodic potential field, the photo-generated charges are separated effectively along the polarization axis. And the photo-generated electrons are transmitted preferentially along the direction perpendicular to the polarization axis, forming a specific two-dimensional transport path, which greatly reduced the e^–^–h^+^ recombination probability. Thus, the activity and selectivity of reducing CO_2_ to CH_4_ are greatly improved. Li's group also found that the surface electric field induced by intrinsic polarity of GaN nanoarrays can effectively enhance carriers’ spatial separation and greatly promote the photocatalytic overall water splitting. Based on the photo-generated charge separation effect between polar and non-polar surfaces, the quantum efficiency was improved from 0.9% to 6.9% with the redox cocatalysts constructed on polar and non-polar surfaces, respectively [56].

Carbon dots synthesized by microwave can quickly extract holes from carbon nitride and prevent the surface adsorption of methanol, which is beneficial to water oxidation and improves the ability of selective CO_2_ reduction to alcohols. Tang and Guo's group found that the carbon dots synthesized by microwave method have unique hole-accepting properties, which can extend the electron lifetime of carbon nitride 6-fold [57]. It is thus beneficial to the stable production of stoichiometric O_2_ and methanol from water and CO_2_, respectively. The selectivity of CH_3_OH is close to 100% and the inherent quantum efficiency is 2.1% under visible light. This work paves the way for the sustainable production of metal-free catalytic methanol, providing a unique strategy that can efficiently and selectively reduce CO_2_ to high-value chemicals via artificial synthesis.

Nanostructured single-crystal photocatalysts with few structural defects and the appropriate cocatalysts were shown to be excellent in overall solar water splitting. For example, although Ta_3_N_5_ photocatalysts have excellent visible light absorption and almost ideal energy band structure, non-single crystal or haploid Ta_3_N_5_ can barely achieve overall water splitting due to the strong charge recombination at defects. Domen's group fabricated Ta_3_N_5_ nanorods growing on lattice-matched cubic KTaO_3_ particles, combined with the Rh/Cr_2_O_3_ cocatalyst. Since the single-crystal Ta_3_N_5_ nanorod crystals had few grain boundaries, the materials presented high water-splitting efficiency under simulated sunlight [33].

Molecular composites are also effective photocatalytic materials for achieving scalable and sustainable carbon dioxide reduction. Reisner's group prepared a photocatalytic sheet by integrating La and Rh co-doped SrTiO_3,_ Mo-doped BiVO_4_, phosphonated Co(ii) bis(terpyridine) and RuO_2_ catalysts onto a gold layer [58] (Fig. 3b). This device achieves a solar energy conversion (from CO_2_ to formate) efficiency of 0.08 ± 0.01% and a selectivity of 97 ± 3%, respectively. When the device was exposed to simulated sunlight, e^–^–h^+^ pairs were produced in both SrTiO_3_: La, Rh and BiVO_4_: Mo. Electrons were transformed from BiVO_4_:Mo conduction band to the SrTiO_3_:La,Rh donor level through the Au layer. With the aid of molecular compounds, e^–^ in SrTiO_3_: La,Rh reduced CO_2_ into HCOO^–^, simultaneously h^+^ in the BiVO_4_:Mo oxidized H_2_O to O_2_ with RuO_2_ cocatalyst for O_2_ generation.

The Z-scheme heterojunction can be propitious to the transfer balance of photo-generated e^–^–h^+^ pairs. Li's group realized the overall CO_2_/H_2_O conversion through marigold-like SiC@MoS_2_ nanoflower materials with 323 μL g^–1^ h^–1^ methane yield and 620 μL g^–1^ h^–1^ O_2_ release under λ ≥ 420 nm visible light irradiation. This photocatalytic performance can be ascribed to the following aspects: (i) the direct Z-scheme heterostructure with negative SiC conduction band for CO_2_ reduction and positive MoS_2_ valence band for O_2_ evolution, (ii) the high e^–^ mobility of SiC and high h^+^ mobility of MoS_2_, (iii) the marigold flower-like microstructure of SiC@MoS_2_ making the surface of catalyst completely exposed to reactants and (iv) the gas–solid reaction beneficial to adsorption/desorption behavior on the Z-scheme heterostructure surface [59] (Fig. 3c).

Through reasonable material design, appropriate doping modification and deposition of cocatalysts, the feasibility of free charge recombination losses for efficient overall water splitting can be achieved. Domen's group demonstrated overall water splitting, evolving H_2_ and O_2_ in a 2 : 1 stoichiometric ratio at an external quantum efficiency up to 96% (350 nm < λ < 360 nm), using Al doped SrTiO_3_ photocatalysts [35]. By selectively photo-depositing the cocatalysts Rh/Cr_2_O_3_ for H_2_ evolution and cobalt hydroxide oxide (CoOOH) for O_2_ generation respectively, the H_2_ and O_2_ evolution could be enhanced separately by anisotropic charge transport on different crystallographic planes of the catalyst particles.

A system with clear structure to clarify the relationship between structure and photosynthesis is very important in promoting the development of artificial photosynthesis. Lan and Liu synthesized metal organic framework (MOF) photocatalysts based on heterometallic Fe_2_M clusters. The catalysts converted carbon dioxide and water into formate and O_2_ without additional sacrificial agents and photosensitizers. Visible light excited heterometallic clusters and photosensitive ligands to produce photo-generated electron-hole pairs. Low-cost metals accepted electrons to reduce carbon dioxide, while high-price metals used holes to oxidize water [60] (Fig. 3d). Lan and Liu's work proposed a novel strategy of designing crystalline catalysts for overall artificial photosynthesis.

The combination of catalysts that are of nanometer and molecular scale is of great significance in visible-light-induced overall artificial photosynthesis. Stolarczyk's group put forward the design idea of ‘all in one’. Spatial separation of oxidation and reduction sites respectively on CdS nanorods and the co-modification parts of the both sites, i.e. Pt nanoparticles and Ru(tpy)(bpy)Cl_2_-based molecules, was realized [31] (Fig. 3e). Pt nanoparticles at the tip of CdS nanorods acted as electron receptors and were responsible for H_2_ production (20 μmol g_cat_^–1^ h^–1^). Ru(tpy)(bpy)Cl_2_-based molecular cocatalysts were fixed to the periphery of CdS nanorods and are responsible for O_2_ production (170 μmol g_cat_^–1^ h^–1^). The catalyst has both reduction and oxidation catalytic functions, so that visible light can drive the total water splitting without sacrificial agents.

Generally speaking, in the last decade, great progress has been made on photocatalytic CO_2_RR. The solar energy conversion efficiency of photocatalytic materials has gradually improved; the methods to improve the photocatalytic reaction efficiency tend to be clear; the understanding of the photocatalytic mechanism has gradually deepened; the characterization methods are developing rapidly; and photocatalytic materials based on novel physical mechanisms are emerging. However, in order to achieve the practical goals of EAP, the research on CO_2_RR still needs a leap forward. First of all, one of the key problems is how to greatly improve the photocatalytic CO_2_RR performance. In addition to the requirement to develop new materials, how to match the band gap of photocatalytic materials with the extraterrestrial solar spectrum, how to match the conduction/valence band position of EAP materials with the potential of reactants, how to reduce electron-hole recombination and improve quantum efficiency, and how to improve the stability of photocatalytic materials are still key scientific issues that must be solved in this field. Secondly, existing characterization techniques cannot fully facilitate an understanding of the catalytic mechanism. Thus, some advanced *in-situ* and/or atomic level characterization methods are still required to reveal the key factors affecting the photocatalytic reaction process. As for realizing efficient and stable EAP, researchers need to deepen the understanding of the photocatalytic reaction mechanism from a macroscopic and qualitative description to microscopic and quantitative research, to comprehensively study the process of light absorption, electron-hole excitation and transport, and interface dynamics, and to clarify the mechanism of energy transfer and conversion. These approaches can guide researchers when it comes to developing EAP materials with high quantum efficiency, by breaking through the existing theoretical framework and actively promoting the cross-integration of photochemistry and other disciplines.

### Photoelectrocatalysis materials for EAP

Photocatalytic overall CO_2_/H_2_O conversion materials are mainly in the form of powders. There are problems such as the undesirable recombination of photo-generated electron-hole pairs in the particles and difficult separation of products from the system. Thus, photoelectrocatalysis, especially non-biased photoelectrocatalysis, in which the catalysts are in the form of film, is more beneficial to the practical applications of EAP.

Photoelectrochemical artificial photosynthesis is generally carried out by photoelectrodes composed of conductive substrates, semiconductors and cocatalysts in the aqueous environment. In this system, the excited photoelectrodes generate electrons or holes that migrate to the surface of the photocathodes or photoanodes for a reductive or oxidation reaction, respectively. Compared with the fact that the charge separation driving force of photocatalysts is the built-in electric field, charge separation in photoelectrocatalytic systems is promoted not only by a built-in electric field but also through external bias. Therefore, some thermodynamically insufficient catalytic reactions can be carried out under photoelectrocatalytic systems with proper bias. In addition, photoelectrocatalysis realizes the spatial separation of the reduction reaction and oxidation reaction to avoid the inverse reaction. According to the module composition of each part of the system, the photoelectrocatalysis system can be divided into a photocathode-to-electrode system, photoanode-to-electrode system, photocathode-photoanode system and photovoltaic coupled photoelectrocatalysis system.

A photocathode usually consists of p-type semiconductors. The conductive band of a p-type semiconductor bends downward at the interface of semiconductor and solution. This band bending allows photo-generated electrons to migrate to the electrode/solution interface and the electrons participate in the CO_2_ reduction reaction to produce hydrocarbon fuel. According to the thermodynamic requirements, the main photocathode materials are Cu_2_O [49], Cu_2_ZnSnS_4_ [50], Co_2_P and p-Si [51].

Correspondingly, a photoanode usually consists of n-type semiconductors. The valence band of an n-type semiconductor bends upward at the semiconductor/solution interface, allowing photo-generated holes to transfer to the electrolyte for O_2_ evolution. The main photoanode materials are TiO_2_, Fe_2_O_3_, WO_3_, ZnO, BiVO_4_, Ta_3_N_5_ and n-Si [3,61–66].

It is usually difficult to construct a device that has an individual photoelectrode and can achieve both CO_2_ reduction and water oxidation conversion for overall artificial photosynthesis (Fig. 1d). The construction of a dual photoelectrode system with both the photocathode and photoanode is beneficial for realizing the full conversion of artificial photosynthesis without bias. The difference in Fermi level between two photoelectrodes determines the theoretical maximum of the photo-generated voltage between them [58,59], which should be larger than the thermodynamic and kinetic requirements.

The photovoltaic coupling system is also used to couple photovoltaic cells to the photoelectrocatalytic system to make effective artificial photosynthetic systems, in which the photovoltaic cell provides bias to assist in driving electrocatalytic reactions. This kind of system includes photovoltaic-photocathode, photovoltaic-photoanode and photovoltaic-electrocatalytic coupling systems (Fig. 1e).

As mentioned above, the photocathode catalysts, photoanode catalysts and photovoltaic electrodes together form the most important material basis in photo-electrocatalytic systems for EAP. Thus, the research progress of photocathode catalysts, photoanode catalysts and photovoltaic electrodes appropriate for EAP will be discussed as follows.

### Photocathode materials

Numerous p-type semiconductors have been exploited and investigated for photocathodes, including p-type silicon [67], oxides [68–73], sulfides [74], phosphides [67] and selenides [75]. Tellurides [76] have been investigated for CO_2_ reduction or H_2_O reduction. In addition, profiting from remarkable CO_2_/H_2_O molecular adsorption and activation, cocatalysts (e.g. Pd, Au, Ag and Cu) [77] usually serve as photocathodes. However, their performance is still limited by high overpotential, low selectivity and long-term operational instability [78]. Rational design is needed to optimize the material interface to achieve efficient charge transfer at low overpotential while maintaining high selectivity.

**Figure 4.**
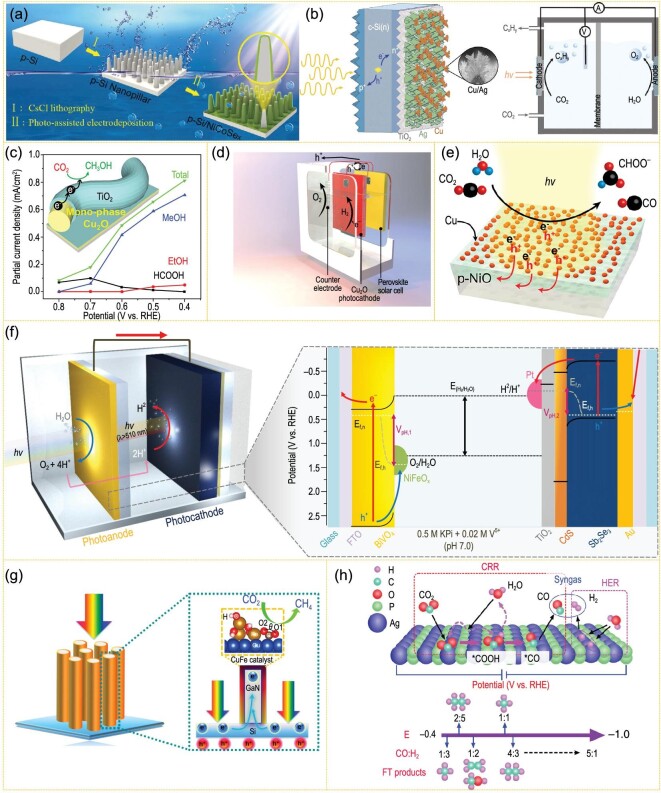
(a) Schematic diagram of the construction process of the p-Si/NiCoSe_x_ photocathode (adapted from ref. [88] with permission from the Royal Society of Chemistry). (b) Left: schematic of the Si/TiO_2_/Ag/Cu photocathode. Right: schematic of the membrane-separated photoelectrochemical cell (PEC) for overall CO_2_/H_2_O conversion (adapted from ref. [90] with permission from the Royal Society of Chemistry). (c) Diagrammatic sketch of the Cu_2_O nanofiber electrode with a Cu_2_O underlayer and a TiO_2_ passivation layer and its CO_2_ photo-reduction performance (adapted from ref. [94] with permission from ACS Publications). (d) A photovoltaic (PV)-PEC system based on CuSCN/Cu_2_O photocathode and perovskite solar cell/IrO_x_ anode for overall H_2_O splitting (adapted from ref. [97] with permission from Springer Nature). (e) Plasmonic Cu/p-NiO photocathodes for CO_2_/H_2_O conversion (adapted from ref. [98] with permission from ACS Publications). (f) Scheme of the NiFeO_x_/Mo: BiVO_4_/FTO||Pt/TiO_2_/CdS/Sb_2_Se_3_/Au/FTO cell for total water splitting (adapted from ref. [100] with permission from Springer Nature). (g) Spatial decoupling of CO_2_ reduction from light absorption and charge separation over CuFe@GaN NWs/Si (adapted from ref. [101] with permission from the National Academy of Sciences, USA). (h) Schematic diagram of selective CO_2_-to-syngas on AgP_2_ (211) (adapted from ref. [102] with permission from Springer Nature).

P-Si is one of the most popular materials for photocathodes, not only because of its high earth reservation, but more importantly, Si has a proper valence band position, with a 1.1 eV band gap. However, its CO_2_ reduction activity is low, and it lacks a suitable cocatalyst to improve the effect. For planar Si electrodes, the overpotential of CO_2_ reduction on the surface is mainly reduced by loading metal cocatalysts [79,80]. Recently, the loading of non-noble metal cocatalysts such as MoS_2_ [81] reduced graphene oxide (rGO) [82], NiO_x_ [83] and Al_2_O_3_ [84] has been found to further improve the stability of Si in the water decomposition process of photoelectrochemical cells (PECs). For example, Bench *et al*. deposited a MoS_2_ thin layer on the surface of an Si photocathode with n^+^p planar structure, and the photocathode could be stable in electrolyte for >100 h [81]. Si nanowires are also widely used in the construction of photocathodes. The main reason is that Si is a typical indirect band gap semiconductor, and its high light absorption efficiency can be ensured when the penetration depth of light reaches 200 μm. The nanowire structure can reduce the migration distance of minority carriers, and multiple reflections of light can ensure the effective absorption of light by Si [85]. Photolithography technology was used to etch and form Si nanopillar arrays on Si wafers, which increased the contact area between photocathode and electrolyte. After Mo_3_S_4_ nanoclusters were deposited on the surface of p-Si, the light absorption range of the material was widened to over 620 nm, and reached a highly increased current density [86]. Recently, Chen *et al*. modified MoS_2_ with band gap position matching p-Si arrays as the cocatalyst, and used it to collect photo-generated electrons to reduce carrier recombination. Therefore, the initial H_2_ evolution potential of an Si@MoS_2_ photocathode is positively shifted to 0.122 V vs. reversible hydrogen electrode (RHE). The researchers further doped MoS_2_ with metal atoms to construct an Si@MMoS_x_ (M = Fe, Co, Ni) photocathode. It was found that the initial H_2_ evolution potential of the material moved positively to 0.192 V vs. RHE after Co doping, and the current density at 0 V vs. RHE reached −17.2 mA cm^–2^ [87]. Jin *et al*. supported a transparent NiCoSe_x_ thin layer on the surface of a bamboo-shoot-like Si nano-array by photo-assisted electrodeposition (Fig. 4a) [88]. Under 100 mW cm^–2^ sunlight intensity, the current density of the catalyst at 0 V vs. RHE reached −37.5 mA cm^–2^. In addition, Hu Xile's research group combined Mo_2_C with amorphous Si and tested it as photocathode in 1 M KOH and 0.1 M H_2_SO_4_. The stable photocurrent density of this material in both electrolytes can reach −11 mA  cm^–2^. This is the first time that the Si-based photocathode has been used in strong alkaline electrolyte, and is also the Si-based photocathode material with the widest pH range so far [89]. Besides, n-type doping on the surface can control the band bending of p-Si and form an n+p depletion layer, thus increasing the photovoltage of the p-Si photocathode. However, the direct contact between n+p-Si and metal catalysts generally causes serious surface recombination and reduced photovoltage. Inserting a metal oxide layer between the Si and catalysts to form a heterojunction can effectively adjust the charge transfer from semiconductor to cocatalysts. CO_2_ reduction in a silicon photocathode was reported to be realized by depositing TiO_2_ through p^+^ implantation and n^+^ implantation on the illumination side, and then loading the Ag/dendritic Cu catalyst. A self-powered CO_2_ reduction device is formed by tandemly coupling the silicon photocathode with two series of translucent CH_3_NH_3_PbI_3_ perovskite solar cells, and the conversion efficiency from sunlight to hydrocarbon and oxygen-containing compounds is 1.5%. Under simulated sunlight conditions, the efficiency of photocathode to hydrocarbons and oxygenated compounds (mainly ethylene, ethanol and propanol) is kept above 60%, which can last for more than several days (Fig. 4b) [90].

Metal oxide semiconductors (such as binary oxide Cu_2_O (2.0 eV)) and ternary oxides (for instance, CaFe_2_O_4_ (1.9 eV), CuNb_3_O_8_ (1.5 eV), CuFeO_2_ (1.5 eV) and LaFeO_3_) are typical p-type semiconductors due to metal vacancies in the structure. Metal oxide semiconductors are extensively utilized for the design and synthesis of photocathode materials because of their easy preparation and low cost. However, their unsatisfactory optical absorption coefficient, carrier mobility and stability make the energy conversion efficiency of oxide photocathodes relatively low [68–73]. Cu_2_O is a typical representative of this type of material and considered as a promising photocathode material to replace Si [91–93]. However, poor stability is the main problem for Cu_2_O-based photocathodes (Fig. 4c) [94]. Grätzel’s group has done a series of work to improve the stability of Cu_2_O-based photocathodes. They used MoS_2+_*_x_* film as a hydrogen evolution reaction (HER) catalyst on the TiO_2_-protected Cu_2_O photocathode, and the current density of the composite electrode could reach −5.7 mA cm^–2^ (0 V vs. RHE) [95]. After that, they deposited a double layer Al :ZnO/TiO_2_ film on the Cu_2_O surface and its improved activity was mainly attributed to the matched conduction band position of Cu_2_O, ZnO and TiO_2_, leading to quick electron migration from electrode to electrolyte [96]. As the best-performing oxide photocathode, the Cu_2_O photocathode’s activity surpasses that of many photocathodes after continuous research and development. However, Cu_2_O photocathodes employing Au as the back contact generally caused considerable e^–^–h^+^ recombination. By employing CuSCN as the h^+^ transport material, h^+^ transport between Cu_2_O and CuSCN is expedited by band-tail states, delivering a 4.55% solar-to-hydrogen (STH) efficiency (Fig. 4d) [97].

p-NiO is also a type of electrode material for CO_2_ reduction PECs. DuChene *et al*. investigated the light-induced modulation of catalytic selectivity over Cu/p-type NiO photocathodes. According to their analysis, the optical excited hot e^–^ of Cu nanoparticles were mainly used for CO_2_ reduction, while hot h^+^ injection from Cu nanoparticles into p-type NiO leads to charge separation. Thus, the optical excitation of plasmonic Cu/p-type NiO photocathodes enhanced CO_2_ reduction and inhibited H_2_ evolution, driving increased production of CO and HCOOH. This work demonstrated a plasmon-driven photocathode for CO_2_ reduction PECs (Fig. 4e) [98].

CuIn_x_Ga_1−x_Se_2_ (CIGS, 1.0–1.68 eV) and Cu_2_ZnSnS_4_ (CZTS, 1.0–1.5 eV) perform outstandingly in the field of solar cells due to their high light absorption coefficient (∼105 cm^–1^) and adjustable direct band gap. The modulation of chemical composition (I = Cu, Ag; II = Al, In, Ga; VI = S, Se, Te) makes the band gap of such a semiconductor adjustable within 1.0–2.4 eV. Furthermore, because of the inherent metal defects (such as Cu vacancy), this type of semiconductors are typical p-type semiconductors, which can be used as a photocathode material. CIGS photocathodes with high current density, such as CuGaSe_2_ (1.7 eV), CuGa_3_Se_5_ (1.8 eV) and CuInS_2_ (1.5 eV), have also been reported in recent years. However, the lattice mismatch at the interface limits the energy conversion efficiency of this kind of material. CdS film prepared by chemical bath deposition (CBD) is one of the best n-type semiconductors reported to compound CIGS to form a p–n junction. However, these materials were difficult in terms of application because of their slow surface reaction process and poor stability in aqueous solution [99]. Therefore, depositing a protective layer on the surface is the most important means to solve the above problems. Sb_2_Se_3_ can also be a promising material for artificial photosynthesis. Yang *et al*. reported an Sb_2_Se_3_ photocathode material with low cost, small band gap and good photoelectric properties and photo-corrosion stability, which also showed a high current density of almost 30 mA cm^–2^ at 0 V vs. RHE. The optimized Sb_2_Se_3_ photocathode achieved 1.5% solar-to-hydrogen efficiency for unassisted water splitting under the condition of 1 sun simulation when combined with a BiVO_4_ photoanode, and the stability exceeded 10 hours (Fig. 4f) [100].

Some traditional cocatalysts (like Ag) are great electrocatalysts for CO_2_-to-CO conversion. However, high overpotential limits the efficiency and the lack of efficient and highly selective cocatalysts restricts the PEC performance of photocathode catalysts. To solve the problem of unsatisfied efficiency resulted from high overpotential, a few ingenious cocatalysts have been designed and present a promising avenue for photocathode catalysts, such as CuFe alloy and AgP_2_. The designed CuFe was reported to exhibit a −38.3 mA cm^–2^ current density with CH_4_ faradaic efficiency up to 51%, resulting in a 2176 h^–1^ turnover frequency using silicon as photocathode under one sun illumination (AM 1.5G) (Fig. 4g) [101]. Integrating AgP_2_ nanocrystal cocatalysts on n^+^p-Si to construct an n^+^p-Si/Al_2_O_3_/AgP_2_ hybrid photocathode and separating the n^+^p-Si and AgP_2_ by the Al_2_O_3_ layer led to a highly improved CO_2_ conversion efficiency. Compared with the Ag cocatalyst, the overpotential of AgP_2_ nanocrystals for CO_2_ reduction to CO is reduced by 0.3 V, and the maximum faradaic efficiency is 82% (Fig. 4h) [102].

### Photoanode materials

Generally, the anodic reaction is an OER in a PEC, which is a vital reaction in extraterrestrial space for human respiration. The photoanode OER is a four-electron transfer process with slow reaction kinetics, which is the rate control step of the artificial synthesis process. Therefore, photoanode materials with high activity and stability are indispensable in improving the energy conversion efficiency of PEC systems for EAP. Numerous n-type semiconductors have been exploited and investigated, including n-type silicon [66], oxides [61–64], nitrides [103] and sulfides [104], as photoanodes for O_2_ production. In addition, finely dispersed cocatalyst nanoparticles (e.g. Co [61], Fe [66]), phosphides [105] and hydroxyapatite [106], or conformal thin layers of cocatalysts (e.g. oxyhydroxide, sulfides) [107] on the photoanode surface are also an effective approach for remarkable O_2_ generation. Among the diverse catalysts, TiO_2_, WO_3_, Fe_2_O_3_ and BiVO_4_ with befitting band structure and plummy stability of aqueous solution, are good n-type semiconductor photoanode materials for O_2_ production through water oxidation [3,61–63]. At present, the O_2_ production of unmodified individual photoanode materials still needs to be improved, mainly due to poor surface reaction kinetics and weak carrier separation and transport. Many studies have improved the above problems by surface modification or defect regulation, design of heterojunction, rational design of cocatalyst, and so forth.

**Figure 5.**
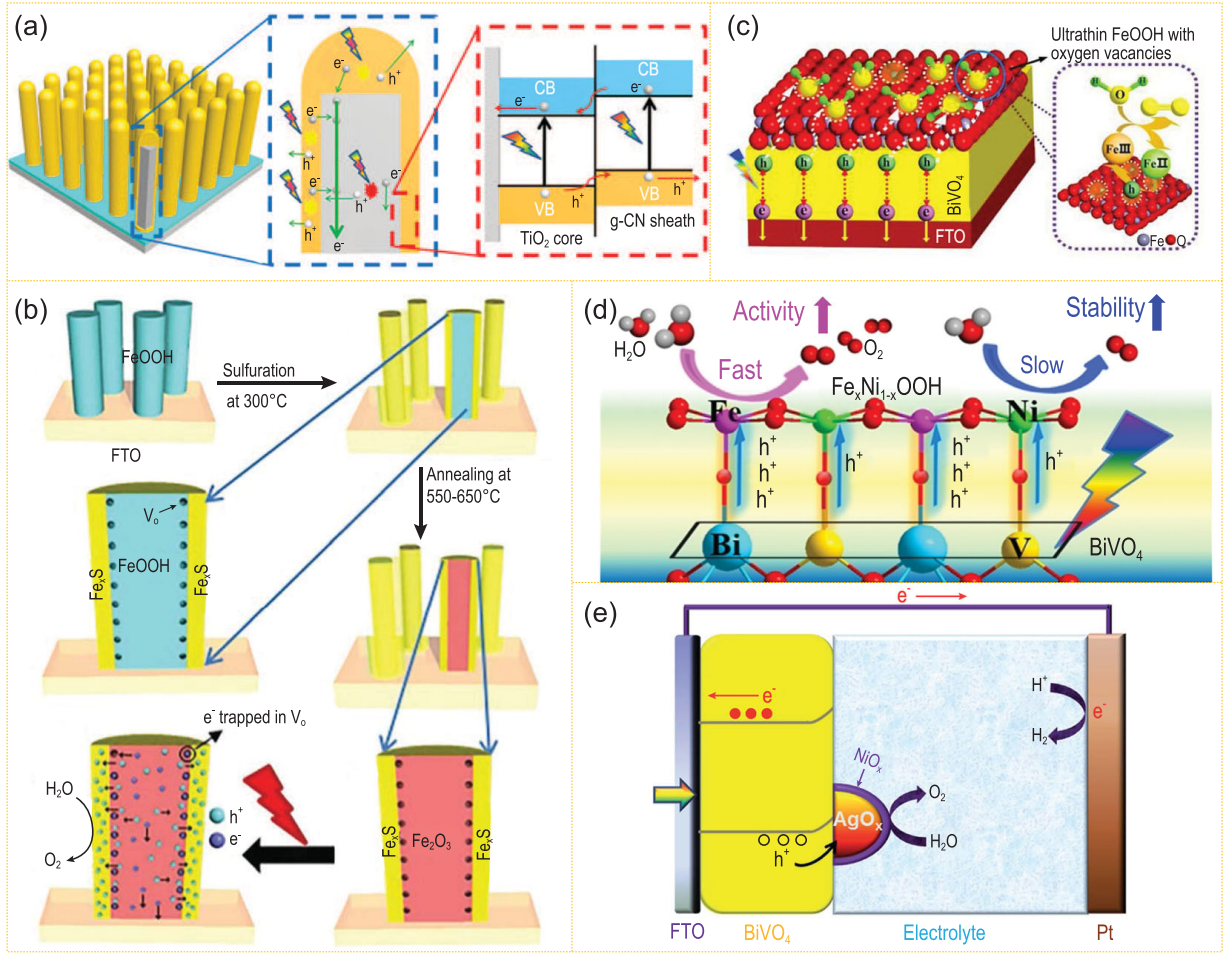
(a) Schematic of the energy diagrams and PEC system of the TiO_2_@*g*-C_3_N_4_ (adapted from ref. [103] with permission from the Royal Society of Chemistry). (b) Diagram of the construction of Fe_2_O_3_/interfacial V_O_/Fe_x_S photoanode and its catalytic mechanism (adapted from ref. [104] with permission from the Royal Society of Chemistry). (c) Illustration of charge transfer during the catalytic process on *β*-FeOOH/BiVO_4_ photoanode (adapted from ref. [110] with permission from Wiley-VCH). (d) Catalytic mechanism on BiVO_4_/Fe_x_Ni_1−x_OOH photoanode (adapted from ref. [111] with permission from Wiley-VCH). (e) Mechanism of solar H_2_O splitting over BiVO_4_/AgO_x_/NiO_x_ photoanode (adapted from ref. [113] with permission from the Royal Society of Chemistry).

Recent research progress on the study of TiO_2_ and BiVO_4_ photoanodes is introduced below. Surface modification and heterojunction construction have been used for improving TiO_2_ photoanode performance. The Ti-OH surface states produced by electrochemical doping on the photoanode of TiO_2_ nanotubes, leading to charge-separation-efficiency increase, contributes to the water oxidation [108]. Heterojunction construction of the g-C_3_N_4_ film on the TiO_2_ nanorod array exhibits obvious advantages in PEC performance (Fig. 5a) [103].

The design of heterojunction and oxygen vacancy defects is demonstrated to be a synergetic method of producing a high performance in photoanodes. Integration of Fe_x_S and the synchronous generation of interfacial oxygen vacancies (V_O_) synergistically reduced the carrier recombination, increased the number of active sites and facilitated the participation of photo-generated holes in water oxidation for the Fe_2_O_3_ photoanode (Fig. 5b) [104]. Lianzhou Wang *et al*. reported a synergetic BiVO_4_ film rich in *in-situ*-formed oxygen vacancy defects converted from Bi_2_S_3_ precursor films through a sulfur oxidation method, in which the electron-hole separation rate of the bulk phase was significantly improved. NiFeO_x_ O_2_-evolution cocatalysts were supported on the photocatalyst surface to facilitate surface O_2_ evolution. A 5.54 mA cm^–2^ photocurrent density was obtained under 1.23 V vs. RHE and simulated sunlight (AM 1.5), with a stability over 80 h. A 6.24 mA cm^–2^ photocurrent density can be obtained by stacking two BiVO_4_/NiFeO_x_ electrodes, and the photoelectric conversion efficiency reaches 2.76% [109]. Bi *et al*. also enhanced the O_2_ production activity of the BiVO_4_ photoanode under a similar mechanism by depositing a 2 nm β-FeOOH film with abundant oxygen vacancies (Fig. 5c) [110].

In addition, for most of the photoanode materials, ingenuity in cocatalyst design has been proven to ameliorate the OER. The selective growth of FeNi cocatalysts on the BiVO_4_ photoanode surface obviously raised the photocurrent density to 5.8 mA cm^–2^ under 1.23 V vs. RHE and simulated sunlight (AM 1.5) (Fig. 5d) [111]. The selective formation of interfacial bonds between Fe, Ni in FeNi cocatalysts and Bi, V on the surface of the BiVO_4_, implied that after optical excitation, Fe-O-Bi interface bonding can effectively transfer the photo-induced holes from BiVO_4_ to Fe active sites. The photo-induced electronic injection will be delivered from Ni atoms to V sites through the Ni-O-V, thus effectively avoiding the V^5+^ ion dissolution. The introduction of Fe species can significantly improve its water-oxidation activity, while Ni can effectively enhance its photoelectric catalytic stability. Inserting black phosphorene (BP) between the OER cocatalyst (NiOOH, MnO_x_ or CoOOH) and BiVO_4_ was reported to improve the PEC performance by 1.2–1.6-fold [112], and a 4.48 mA cm^–2^ photocurrent density at 1.23 V vs. RHE was achieved by the NiOOH/BP/BiVO_4_ photoanode. The intrinsic p-type BP can enhance h^+^ extraction and the h^+^ trapping lifetime on the BiVO_4_ surface, while the OER cocatalyst overlayer can suppress catalyst self-oxidation for achieving a high durability. This research presents an advantageous nexus between cocatalyst and semiconductor. AgO_x_/NiO_x_ composite cocatalysts can be exploited to hoist the H_2_O oxidation kinetics, as well as the carrier separation of BiVO_4_ photoanodes, because of the high-valence-state stabilization of the metal ions, the formation of H_2_O oxidation active sites and the extension of the band bending region induced by AgO_x_/NiO_x_ (Fig. 5e) [113].

### Photovoltaic (photo)electrocatalytic materials

Compared with powder photocatalysis, photoelectrocatalysis constructs the macro-space separation of oxidation and reduction half reactions, which is beneficial to the separation of catalytic products. However, for higher catalytic performance, photoelectrocatalysis depends on the input of external electric energy. Recently, photovoltaic cells have been applied for photoelectric conversion, and the photovoltaic-electrocatalytic CO_2_/H_2_O conversion technology has also been greatly developed. Photovoltaic (photo)electrocatalytic overall CO_2_/H_2_O conversion with high efficiency provides an effective avenue for storing solar energy, which can greatly benefit EAP.

Solar cells are mainly categorized into silicon-based, III–V-based or perovskite-based photovoltaic electrocatalytic devices [114]. As high operational current densities are demanded in photovoltaic electrocatalytic devices, high-quality electrocatalysts (e.g. Au, Pt, IrO_x_) are preferred. Commercial noble-metal-based electrodes such as IrO_2_ (for O_2_ evolution) and Au (for CO_2_ reduction) [115] were successfully used for overcoming the sluggish kinetics of CO_2_ reduction and O_2_ evolution, respectively, which hinder the extensive implementation of overall CO_2_/H_2_O conversion. However, the moderate performance limits the total efficiency, and resource scarcity increases the cost. Therefore, taking further consideration of economy, resource abundance and high product selectivity, more cost-effective materials, such as Fe, Ni, Zn, W, Cu, Co or Ti-based materials, are research hotspots. The products of artificial photosynthesis are developed gradually to obtain non-toxic fuels rather than CO.

**Figure 6.**
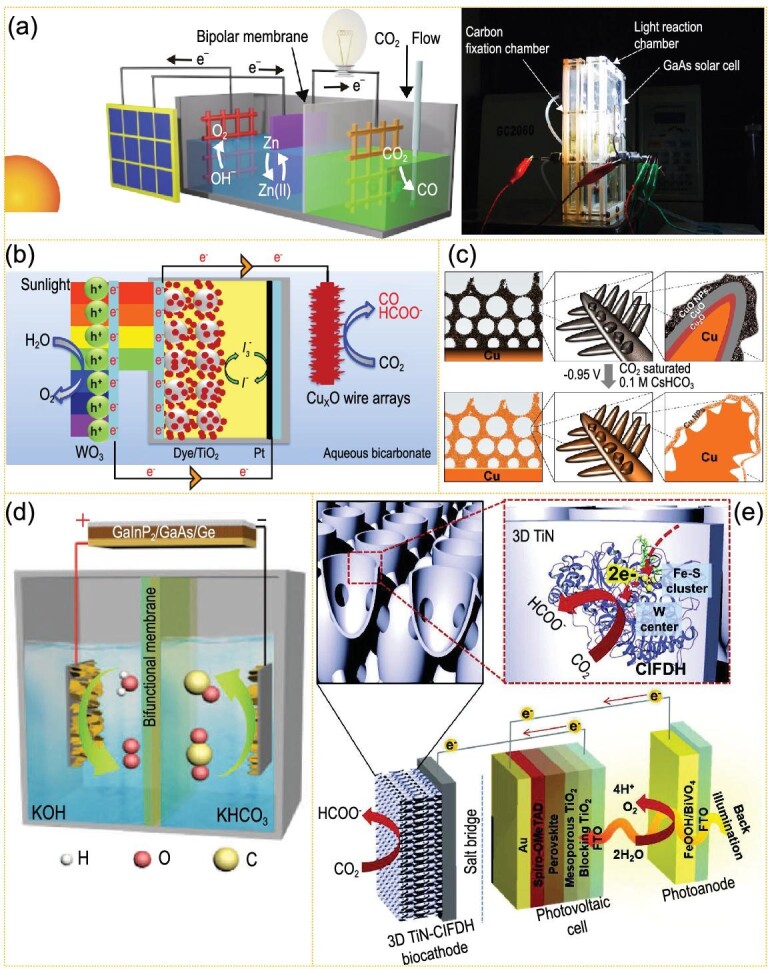
(a) Schematic and photograph of the redox-medium-assisted CO_2_ photovoltaic electrocatalytic reduction system containing a nickel-iron hydroxide electrode and a zinc/zincate redox with a gold nanocatalyst (adapted from ref. [116] with permission from Springer Nature). (b) Illustration of a WO_3_ (photoanode)||Cu_x_O (photocathode) system coupling with a dye-sensitized solar cell (adapted from ref. [117] with permission from Elsevier). (c) Schematic view of the dendritic nanostructured CuO material before (top) and after (bottom) CO_2_ photovoltaic electrocatalytic reduction in 0.1 M CsHCO_3_ (adapted from ref. [118] with permission from the National Academy of Sciences, USA). (d) Illustration of the solar-driven overall CO_2_ splitting system with Co_2_FeO_4_ nanoarrays both as anode and cathode (adapted from ref. [119] with permission from Wiley-VCH). (e) Schematic illustration of the BiVO_4_/perovskite/3D TiN-ClFDH biocatalytic tandem PEC system for unbiased, solar-driven formate production (adapted from ref. [120] with permission from Wiley-VCH).

Recently, Zheng *et al*. constructed a GaAs solar cell with a nickel-iron hydroxide electrode for H_2_O oxidation, and Au nanocatalysts to reduce CO_2_ to CO, between which a Zn/Zn^2+^ redox medium was used for optimized charge transfer. This redox medium auxiliary system can achieve 15.6% solar energy-carbon monoxide photoelectric conversion efficiency and 63% electric energy efficiency under one sun intensity (Fig. 6a) [116]. Park *et al*. constructed a WO_3_ photoanode and Cu_x_O photocathode system coupled with a dye-sensitized solar cell for driving CO_2_ reduction on Cu_x_O and water oxidation on WO_3_ with zero external bias. In this dual-light-absorbing cell, the solar-to-chemical energy efficiency of CO_2_ reduction for CO is 2.5%, while it is 0.7% for H_2_ and 0.25% for HCOOH, respectively (Fig. 6b) [117]. By using Cu-based catalysts at both anode and cathode coupled with a perovskite photovoltaic mini-module, Fontecave *et al*. reported a 21% energy efficiency and a 2.3% solar-to-hydrocarbon efficiency of CO_2_ reduction to CH_2_=CH_2_ and CH_3_CH_3_ (Fig. 6c) [118]. By using Co_2_FeO_4_ nanosheet arrays for both cathode and anode, driven by a GaInP_2_/GaAs/Ge photovoltaic cell, Cao *et al*. showed a complete overall CO_2_/H_2_O conversion system with 13.1 mA cm^–2^ photocurrent density, corresponding to 15.5% solar-to-CO efficiency (Fig. 6d) [119]. The dual functional attributes can be attributed to the formation of ^*^COOH and ^*^O intermediates that originated from the Co sites in Co_2_FeO_4_. Lee *et al*. reported an enzyme—W-containing formate dehydrogenase (FDH) from *Clostridium ljungdahlii* (ClFDH)-conjugated direct electron transfer-type biocathode based on TiN nanoshell—and applied it to a PV-PEC system with free bias, which showed promising and stable solar-driven CO_2_-to-HCOOH conversion at a rate of 0.78 μmol h^–1^ for 24 h and a 77.3% faraday efficiency (Fig. 6e) [120].

Although the research history of the photoelectrocatalytic CO_2_ reduction reaction has been developed over tens of years with great progress, there are still many problems and challenges. Because of the low catalytic efficiency, it is far from meeting the requirements of EAP application:

- The selectivity of high carbon products is still very low. In CO_2_RR, although the faraday efficiency of CO and HCOOH can reach >90%, the highest faraday efficiencies of other reduction products, which are much more desired for EAP technology, such as CH_4_, CH_3_OH, C_2_H_5_OH, CH_3_COOH and C_2_H_4_, are only at the level of ∼50%.
- The catalysts have poor stability. Stability is an important parameter to characterize the advantages and disadvantages of a catalyst material, and it is also a problem that must be overcome to realize EAP in harsh extraterrestrial environments. Although some catalytic materials remain stable for hundreds of hours, they are still far from the EAP goals. In the field of photoelectrocatalysis especially, due to the complex structure of photocatalysts/cocatalysts, high-energy radiation corrosion and other factors, the stability of photoelectrocatalytic CO_2_RR is a great challenge to overcome.
- The reaction process of (photo)electrocatalytic CO_2_RR requires a deeper understanding. Light absorption, charge separation and interfacial reaction in the process of (photo)electrocatalytic CO_2_RR not only have differences on the time scale, but are also often localized at the atomic or molecular level on the spatial scale. Therefore, for observing the (photo)electrocatalytic CO_2_RR process in order to provide a reference for future theoretical analysis, transient, microregion and *in-situ* analysis methods should be utilized and developed.

With regard to the selection of the most suitable EAP technology, photocatalysis, photoelectrocatalysis and photovoltaic electrocatalysis have their individual advantages and disadvantages. EAP based on photocatalysis without an external complex device system is portable and easy to work, and therefore more adaptable to the complex extraterrestrial environment. However, its low reaction efficiency leads to low concentration of the products, with difficulties in separation and enrichment for CO_2_RR and OER products. Photoelectrocatalysis with two-electrode systems has the obvious advantage of efficient product separation. However, the very low stability of semiconductor photoelectrocatalysts under high-energy radiation makes the photoelectrocatalytic process difficult to adopt currently. In comparison, the photovoltaic coupled with electrocatalysis process can realize energy storage by separating photoelectric conversion and energy-to-chemical conversion, thus being more suitable for application in the extraterrestrial environment in recent times. With the further development of semiconductor/cocatalyst materials, we believe that composite technology with two or more systems will be the trend of EAP applications. More specific and detailed information for each system can be seen in Table 1.

**Table 1.** More specific and detailed information about the example references.

Method	Catalyst	Reactant	Light source/intensity	Current densities	Product/selectivity	TOF	Efficiency	Ref.
Photocatalysis	4.5(ZnGa_2_O_4_):(Zn_2_GeO_4_)	CO_2_, 0.4 mL deionized water	A UV-enhanced (200 to 350 nm) 300 W xenon arc lamp	-	0.5 μmol h^–1^ CH_4_, 3 μmol h^–1^ O_2_	-	-	[52]
	CeO_2_ octahedron textured with nanorods	CO_2_, 0.4 mL deionized water	300 W Xe lamp (full spectrum)	-	0.86 μmol h^–1^ g^–1^ CH_4_, ∼2 : 1 molar ratio of O_2_ to CH_4_	-	CH_4_ quantum efficiency (%) = 0.2% at 380 nm	[54]
	Rh-CoO_x_/GaN nanorod arrays	Pure aqueous solution	300 W Xe lamp (full spectrum)	-	H_2_ and O_2_ in stoichiometric ratio (2 : 1)	-	OER quantum efficiency (incident light wavelength range of 250–400 nm) reaches 81.1%, quantum efficiency of overall water splitting reaches 6.9%	[56]
	Microwave-synthesized carbon-dots decorated carbon- nitride	CO_2_, 10 mL water	300 W Xe lamp (λ > 420 nm)	-	Methanol 13.9 μmol h^–1^ g^–1^_,_ CO 0.05 μmol h^–1^ g^–1^_,_ O_2_ to methanol is ∼1.45 : 1; 99.6% selectivity from CO_2_ to methanol	-	Internal quantum efficiency (IQY) of 2.1% in the visible region	[57]
	CotpyP-loaded SrTiO_3_:La,Rh|Au|RuO_2_-BiVO_4_:Mo photocatalyst sheet	CO_2_-saturated 0.1 M KHCO_3_	AM 1.5 G (1 sun) illumination (100 mW cm^–2^)	-	O_2_ 1.3 μmol h^–1^ cm^–2^, HCOO^–^ 16.1 μmol in 5 h	TON for HCOO^−^ was 305 after 4 h	A solar-to-formate conversion efficiency of 0.08 ± 0.01% with a selectivity for formate of 97 ± 3%	[58]
	SrTiO_3_:Al photo-deposited with Rh/Cr_2_O_3_/CoOOH		Xe lamp (300 W, full arc)	-	Overall water splitting, evolving H_2_ and O_2_ in a 2 : 1 stoichiometric ratio	-	An IQE of 100% and an external quantum efficiency (EQE) >50%	[35]
Photoelectrochemical cells	Si@CoMoS_x_ photocathode	0.5 M H_2_SO_4_	500 W xenon lamp equipped with an AM 1.5 filter (100 mW cm^–2^)	−17.2 mA cm^–2^ at 0 V_RHE_	H_2_ 3.86 μmol min ^–1^	-	Faradaic efficiency 81.0%	[87]
	p-Si/NiCoS_x_ core/shell NP arrays	0.5 M H_2_SO_4_	AM 1.5G illumination (100 mW cm^–2^)	-37.5 mA cm ^–2^ at 0 V_RHE_	H_2_	-	-	[88]
	a-Si/Mo_2_C	0.1 M H_2_SO_4_ and 1.0 M KOH	AM 1.5G illumination (100 mW cm^–2^)	-11.2 mA cm^–2^ at 0 V_RHE_	H_2_	-	-	[89]
	An Si photocathode with two series-connected CH_3_NH_3_PbI_3_ perovskite solar cells	0.1–0.5 M CsHCO_3_	AM 1.5G illumination (100 mW cm^–2^)	No bias	Ethylene, ethanol and 1-propanol	-	Total solar-to-chemical conversion efficiency of 3.5% to all products and 1.5% to hydrocarbons and oxygenates	[90]
	MoS_2+_*_x_* film as the HER catalyst on TiO_2_-protected Cu_2_O photocathode	1 M Na_2_SO_4_ buffered with 0.1 M K_3_PO_4_ (pH = 5.0)	AM 1.5G illumination (100 mW cm^–2^)	−5.7 mA cm^–2^ at 0 V_RHE_ (pH 1.0)	-	-	7% STH efficiency in a tandem cell configuration	[91]
	Cu_2_O photocathode using solution-processed CuSCN as hole transport material	0.5 M Na_2_SO_4_ (pH = 5.0)	AM 1.5 G illumination (100 mW cm^–2^)	6.4 mA cm^–2^ at 0 V_RHE_	-	-	STH efficiency of 4.55%	[97]
	Sb_2_Se_3_ photocathode with a BiVO_4_ photoanode	0.5 M KPi buffer + 0.01 M V_2_O_5_ (pH = 7.0)	AM 1.5 G illumination (100 mW cm^–2^)	-	-	-	STH efficiency of 1.5% with stability over 10 h	[100]
	CuFe catalyst coupled with GaN nanowires on n^+^-p silicon wafer	CO_2_-purged 0.5 M KHCO_3_ aqueous solution (pH ≈ 8.0)	AM 1.5 G illumination (100 mW cm^–2^)	−38.3 mA cm^–2^ at −1.2 V_RHE_	CH_4_ 88.8 μmol h^–1^ cm^–2^	2176 h^–1^	Faradaic efficiency of 51%	[101]
	A photocathode consisting of a n^+^p-Si wafer coated with ultrathin Al_2_O_3_ and AgP_2_ nanocrystals	CO_2_-saturated 0.5 M KHCO_3_ solution	AM 1.5 G illumination (100 mW/cm^2^)	−3.2 mA cm^–2^ at −0.11 V_RHE_	-	-	FE_CO_ of 67% at −0.2 V vs. RHE	[102]
	BiVO_4_/NiFeO_x_	1 M borate buffer electrolyte containing 0.2 M Na_2_SO_3_	AM 1.5 G (100 mW cm^–2^)	5.54 mA cm^–2^ at 1.23 V_RHE_	-	-	Photon-to-current efficiency of 2.76%	[109]
	Ultrathin FeOOH nanolayers with abundant oxygen vacancies on BiVO_4_ photoanode	0.2 m Na_2_SO_4_ (pH = 7.0)	AM 1.5 G illumination (100 mW cm^–2^)	4.3 mA cm^–2^ at 1.23 V_RHE_	H_2_ 92.3 μmol after 120 min	-	-	[110]
	BiVO_4_/Fe_x_Ni_1−x_OOH photoanode	0.5 m K_3_BO_3_ electrolyte (pH = 9.5)	AM 1.5 G (100 mW cm^–2^)	5.8 mA cm^–2^ at 1.23 V_RHE_	H_2_ 388 μmol, O_2_ 172 μmol after 3 h	-	The average incident photon to current efficiency (IPCE) of 90% at 420 nm	[111]
	NiOOH/BP/BiVO_4_ photoanode	0.5 M phosphate buffer (KPi, pH = 7.1)	AM 1.5 G (100 mW cm^–2^)	4.48 mA cm^–2^ at 1.23 V_RHE_	-	-	-	[112]
	BiVO_4_/AgO_x_/NiO_x_ photoanode	0.2 M KBi aqueous solution (pH = 9.25)	AM 1.5 G illumination (100 mW cm^–2^)	1.24 mA cm^–2^ at 0.6 V_RHE_		-	The average IPCE values in the 380–450 nm range increased from 22.4% to 49.3%.	[113]
Photovoltaic cell + electrocatalysis	A photovoltaic and electrocatalytic system consisting of GaAs solar cell, nano-Au catalyst cathode and NiFe hydroxide anode and Zn/Zn(II) redox medium	0.5 M CO_2_-saturated KHCO_3_ (pH = 7.2)	AM 1.5 G illumination (100 mW cm^–2^)	10 mA cm^–2^ at 1.96 V_RHE_	-	-	FE_co_ of ∼92%, a solar-to-CO photo-conversion efficiency of 15.6%, and an electric energy efficiency of 63%	[116]
	A PEC composed of WO_3_/dye-sensitized solar cell and Cu_x_O (*x *= 1 and 2) wire arrays as a dual-absorber photoanode and cathode	CO_2_-purged 0.1 M bicarbonate aqueous solution	AM 1.5 G illumination (100 mW cm^–2^)	-	CO, H_2_ and formate	-	The primary CO_2_ conversion product is CO, with a solar-to-chemical energy efficiency of ∼2.5%; H_2_ and formate are obtained with energy efficiencies of 0.7% and 0.25%, respectively in 5 h (overall efficiency ∼3.45%)	[117]
	A system coupling a photovoltaic cell to an electrochemical cell using the dendritic nanostructured copper oxide material at both the anode and cathode	Cathodic electrolyte: 0.1 M CO_2_-saturated CsHCO_3_ (pH = 6.8); anodic electrolyte: 0.2 M Cs_2_CO_3_ (pH = 11)	Without external bias and AM 1.5 G (1 sun) illumination (100 mW cm^–2^)	A stable current of 6.0 ± 0.2 mA and a potential of 2.8 ± 0.02 V	C_2_H_4_, C_2_H_6_, CO, HCOOH, H_2_	-	C_2_H_4_ and C_2_H_6_ as the main products with an average faradaic yield (FY) of 40.5% (34% for C_2_H_4_ and 6.5% for C_2_H_6_), together with CO and HCOOH in 4.8% and 6.4% FY, respectively Concomitant H_2_ production was 42.2% FY	[118]
	GaInP/GaAs/Ge/Co_2_FeO_4_ driven by a triple junction GaInP_2_/GaAs/Ge photovoltaic cell	0.1 M CO_2_-saturated KHCO_3_	AM 1.5 G illumination (100 mW cm^–2^)	13.1 mA cm ^–2^ at −1.2 V_RHE_	-	CO	Solar-to-CO efficiency of 15.5%	[119]
	W‐containing formate dehydrogenase from *Clostridium ljungdahlii* (ClFDH) on the 3D TiN nanoshell	CO_2_ saturated phosphate buffer	Xe lamp (photon energy flux of 100 mW cm^–2^, 420 nm cut‐off filter)	Bias‐free	Formate 0.78 μmol h^–1^	-	Faradaic efficiency 77.3%	[120]

## CONCLUSION AND PERSPECTIVE

In summary, the development of EAP materials is highly prospected. Looking at the future of space science and technology, how to achieve extraterrestrial survival and affordable and sustainable deep space exploration through EAP technology under extreme conditions, has become the common pursuit of humanity. On this frontier, there are still a series of concomitant scientific problems, such as low concentrations of CO_2_/H_2_O, different solar radiation intensities, ultrahigh gravity or microgravity, extreme temperatures, intense cosmic radiation, extreme pressure and ultimate vacuums. The problems that urgently need to be resolved are listed below:

- CO_2_/H_2_O photo-conversion materials with normal working ability at low atmospheric density are the linchpin for EAP. At present, research into CO_2_/H_2_O photo-conversion mainly focuses on atmospheric conditions with a high concentration of, or pure, CO_2_. However, the concentration of CO_2_ or H_2_O in the extraterrestrial environment or the enclosed space inside the capsule is usually at an ultralow level or unstable state. Therefore, how to realize the enrichment and further utilization of CO_2_ is one of the critical problems. Development of artificial photosynthetical materials in low CO_2_ concentration environments or CO_2_ collection technology may be considered as the solutions.
- It is necessary to develop a photocatalytic material system suitable for withstanding different solar radiation intensities, strong cosmic radiation and Frenkel defects under extraterrestrial conditions. Due to the difference in solar radiation intensity and spectral distribution between outer space and the Earth’s surface, photocatalytic materials developed for the solar spectral conditions on Earth may not work effectively and stably in the outer space environment. Therefore, it is necessary to develop new artificial photosynthetic materials suitable for cosmic radiation with a long working life, high stability, wide spectrum and responsiveness to AM0 spectra, to meet the requirements of long-term space exploration tasks. Ceramics or composites can be selected to achieve thermostability and thermal shock resistance. Metal oxides such as PbO, BaO and Bi_2_O_3_ with a high atomic number, or rare metallic elements, can be added to the material for radiation resistance [121].
- Research into the effects of extreme temperature, ultra-vacuum and microgravity on the photochemical reaction, as well as multi-photon processes in the photochemical reaction, will give clues as to how to plan the EAP process. The increase of interfacial resistance (ohmic drop) caused by bubbles produced in the photocatalytic process will severely affect the surface coverage of electrodes. The mass transfer process under microgravity will become more difficult, greatly reducing the energy efficiency of the system. Supersaturated gas layers formed by gas reactants and products gathered near the three-phase interfaces of electrodes have a very important negative influence on the reaction process, material transport and reaction efficiency. Therefore, it is necessary to deeply explore the diffusion and transfer process of reaction media in electrolyte under microgravity, as well as the key mechanisms of bubble nucleation, growth, interface separation, gas-liquid two-phase flow and gas-liquid separation and their influence on photocatalytic processes. In addition, the state-of-the-art technologies of ECLSS architecture as a subsystem typical of a crewed space vehicle, which provides all the necessary conditions, can be used for the control of steady temperature and pressure inside the space capsule to support the artificial photosynthetic material, devices or systems.
- The photo/thermo/electric coupling catalysis mechanism needs to be deeply understood and realized by utilizing the full spectrum of solar energy, 99.9% of which is in the infrared region (43%), visible region (50%) and ultraviolet region (7%). The band gap of existing photocatalytic materials is extensively large, and most of them absorb the ultraviolet or near-ultraviolet spectrum. As a result, the utilization rate of solar energy is not high and the overall efficiency of photocatalysis is relatively low. It is necessary to explore the mechanism of solar photo/thermo/electric coupling catalysis, develop a composite photocatalytic system with multi-spectral absorption and full-spectral utilization, and improve the reaction rate of photosynthesis and photochemical conversion efficiency.
- Recently, many new artificial photosynthesis processes by microorganism/semiconductor composite systems have been developed. The core problem of the artificial photosynthesis system based on microorganisms is the interaction between microorganisms and inorganic materials, the key of which lies in the transfer of energy and charge at the interface. The interface interaction not only affects the expression of microorganisms, but also has an important impact on the properties of materials. Therefore, it is very important to improve the solar energy to chemical energy conversion efficiency of artificial photosynthesis systems based on microorganisms by increasing the interface charge transfer rate.
- Advanced *in-situ* and atomic-scale analysis, and computational simulation techniques will play an important role in EAP. Through advanced *in-situ* micro-analysis and computational simulation, scientists can fully understand the complex reaction process and intermediate products in CO_2_/H_2_O photo-conversion, and thus help overcome the obstacles in energy efficiency, reaction selectivity and total conversion rate.

How to convert CO_2_ from human respiration into O_2_? How to use CO_2_/H_2_O on Mars or in other extraterrestrial atmospheric environments to generate O_2_ and fuel? These are the core missions of human beings with regard to achieving extraterrestrial survival and sustainable exploration. In recent years, driven by sustainable development on Earth, the technology of artificial photosynthesis has rapidly developed. In space exploration activities, it will become a core ability to *in-situ* transform CO_2_/H_2_O at room temperature into the basic material needed for human beings to survive outside the Earth. Chinese scientists put forward the concept of EAP, took the lead in developing artificial photosynthesis devices and space experiments that will greatly promote the development of this field, and will guide research in the fields of materials, physics, chemistry, energy, aerospace science and technology.
